# Integrated transcriptomic and metabolomic analyses reveal optimized cultivation strategies for purple potatoes (*Solanum tuberosum* L.)

**DOI:** 10.3389/fpls.2025.1675151

**Published:** 2025-12-01

**Authors:** Weiran Zhong, Jin Yang, Fengming Liang, Qiang Ma

**Affiliations:** 1 Corn and Characteristic Crops Research Institute, Chongqing Academy of Agricultural Sciences, ChongQing, China; 2 Wuxi Shuguang Agricultural Technology Development Co., Ltd., Wuxi, China

**Keywords:** purple potato, basal fertilizer, plant density, anthocyanin, starch

## Abstract

Potato (*Solanum tuberosum* L.), a cornerstone of global food security, faces significant yield limitations worldwide. Fertilization regimes and planting density critically determine potato yield and quality. This study integrated transcriptomic and metabolomic analyses to investigate the effects of basal fertilizer rates and planting density on tuber yield and quality in the purple cultivar ‘Jinyun No. 5’, aiming to establish sustainable high-efficiency cultivation protocols for pigmented potatoes. In the mountainous areas of southern China, the optimal combination (0.15 kg·m^-^² basal fertilizer and 5.25 plants·m^-^²) significantly enhanced yield, anthocyanin, and starch content. Yield increased by 12.9% versus minimum density (3.75 plants·m^-^²) at equivalent fertilization, and by 6.6% versus minimum fertilization (0.05 kg·m^-^²) at equivalent density. Metabolomic profiling revealed marked accumulation of flavonoids and phenolic acids under optimal conditions, while transcriptome sequencing identified upregulation of key genes involved in anthocyanin biosynthesis (ANS, DFR) and starch metabolism (SBEI, SBEII). These findings provide molecular insights into how optimized cultivation strategies improve both yield and nutritional quality in purple potatoes.

## 1 Introduction

Potato (*Solanum tuberosum* L.), a globally vital staple crop, ranks as the world’s fourth most consumed food crop after rice and wheat, sustaining over a billion people (Fao publications catalogue 2021, 2021). Characterized by high yield potential, nutritional richness, adaptability, and economic importance, potatoes are consumed both fresh and processed, playing a critical role in global food security and sustainable agriculture (Zhang et al., 2017; Nurmanov et al., 2019). China leads global potato production (25% of total output), yet achieved only 16.3 t·ha^-^¹ in 2021, below the global average (20.7 t·ha^-^¹) (Fao publications catalogue 2023, 2023).This yield gap highlights significant potential for enhancing potato productivity (Sun, 2010).

Modern potato cultivation integrates traditional agronomy with technological innovation to enhance yield and quality. Key yield determinants include seed quality, climate, irrigation, fertilization regimes, and pest/disease management (Luo et al., 2024; Tessema et al., 2024). Among these, suboptimal fertilization and planting density critically constrain productivity (Yin et al., 2023). China’s potato cultivation faces unique challenges: its notably dispersed and small-scale operations (compared to other crops) complicate systematic regional strategy development for cultivation and nutrient management (Wang et al., 2023). In southern mountainous regions—where potatoes serve as a staple food—irregular terrain and poor soil conditions significantly reduce yields. Due to low-quality arable land, crops depend heavily on exogenous fertilizers for essential nutrients, making application rates direct determinants of yield and quality (Milroy et al., 2019; Assunção et al., 2021). Therefore, increasing tuber yield per unit area and improving fertilizer-use efficiency are imperative to minimize resource waste, reduce environmental pollution, enhance economic returns, and ensure sustainable development of the potato industry (Ginter et al., 2023; Jiang et al., 2025).

Nitrogen (N), phosphorus (P), and potassium (K) are essential macronutrients for potato development. Optimal application increases starch, soluble protein, and Ascorbic Acid content while lowering reducing sugars and improving tuber quality (Guo et al., 2022). Decadal field trials establish an optimal N range of 150–225 kg·ha^-^¹ with N: P: K ratios of 1:0.3–0.5:0.8–1.2 (Gumul et al., 2020) (Lachman et al., 2009). When potatoes are grown under nitrogen-deficient conditions, it will lead to a significant reduction in crop height, chlorophyll content, dry matter, and nitrogen accumulation (Liu et al., 2015; Guo et al., 2022) (Ezekiel et al., 2011). Planting density significantly influences the construction and optimization of a reasonable population structure in potato plants, serving as the most critical factor affecting yield per unit area (Brown et al., 2005; Guo et al., 2024; Ning et al., 2024). When combined with optimal fertilization, it enhances tuber size, commercial quality, and starch accumulation (Xiao et al., 2023; Wu and Xiao, 2024).

Compared to conventional cultivars, purple-fleshed potatoes exhibit superior nutritional profiles due to elevated polyphenols—notably anthocyanins (97.05–104.03 mg cyanidin-3-glucoside equivalents/100 g DW) and phenolic acids with demonstrated antioxidant activity (Sun et al., 2023; Yuan et al., 2024). Recent advances in omics technologies have facilitated a more comprehensive understanding of the molecular mechanisms underlying quality traits in pigmented potatoes. Liu et al. identified differential expression of anthocyanin biosynthetic genes in tetraploid purple- versus white-fleshed potatoes (Liu et al., 2015).Anthocyanins belong to an important subclass of flavonoids, and their biosynthetic pathway is a branch of flavonoid metabolic pathway. It contains several key enzymes and genes: Dihydroflavonol is reduced to colorless anthocyanins by dihydroflavonol reductase (DFR), oxidized to colored anthocyanidins by anthocyanin synthase (ANS), and finally modified to stable anthocyanins by glycosyltransferase (UFGT). Purple potato pomace (starch processing byproduct) contains 17.05 g dietary fiber/100 g DW (13.4 g insoluble, 3.65 g soluble), supporting gastrointestinal health and glycemic control (Ezekiel et al., 2011; Gumul et al., 2020). These beneficial compounds, which are not present in white potatoes, may reduce oxidative stress and inflammation, potentially mitigating the onset of chronic diseases (Brown et al., 2005). Metabolomic studies have further characterized the diverse profiles of flavonoids and phenolic acids in purple potatoes, which are closely associated with their enhanced antioxidant capacity (Xiao et al., 2023; Wu and Xiao, 2024). Collectively, these foundational studies highlight the importance of integrating transcriptomic and metabolomic approaches to elucidate the regulatory networks governing nutrient accumulation in pigmented potatoes.

RNA sequencing (RNA-seq) can analyze the gene expression levels of plant tissues under specific conditions, which helps to understand the molecular mechanisms of physiological indicators, discover candidate genes, and is a fundamental method for studying crop traits such as yield or stress resistance (Wang et al., 2009). We systematically assessed the effects of fertilizer application rates and planting densities on yield and key nutritional compounds, with RNA-seq and metabolomics serving as the principal analytical methodologies (Liu et al., 2015; Guo et al., 2022). Utilizing nutritionally enhanced purple potatoes (*Solanum tuberosum* L.) as experimental material, this study established optimized fertilization and planting density protocols to achieve sustainable high-efficiency cultivation of pigmented potatoes.

## 2 Materials and methods

### 2.1 Plant materials and growth conditions

Purple potato (Solanum tuberosum L.), specifically Jinyun No.5 (cultivar ‘C21’), was used as experimental material. It was cultivated in the field under normal farming practices and management at Chongqing Academy of Agricultural Sciences. C21 is a colorful fresh-food type, averages 3.4 main stems per plant, and exhibits high resistance to late blight. The soil in the experimental field had a pH of 4.47, with organic matter content at 29.7 g/kg, alkali-hydrolyzable nitrogen(N) (i.e., water-soluble nitrogen) at 151 mg/kg, available phosphorus(P) at 172 mg/kg, and available potassium(K) at 124 mg/kg.

The plants were planted in a completely randomized design with three replications. Each plot covered an area of 1.00 m² and contained 6 plants. The double-row ridge planting system was adopted, with two wide ridges per plot. The ridges were spaced 100 cm apart, each measuring 5.33 m in length. Narrow rows within ridges were spaced 25 cm apart, and plant spacing was set at 33 cm. Sowing occurred on January 12, 2024, and harvesting occurred on June 14. Climatic parameters were those recorded in Chongqing, China during this period (**Supplementary Table 1**). The average monthly temperature ranged from 4.4°C in January to 21.1°C in June, with total precipitation during the growing season amounting to 862.7 mm. Precipitation exhibited substantial intermonthly variation, including 78.3 mm in January and 235.3 mm in April. A drip irrigation system was implemented to maintain soil moisture at 70–80% of field capacity throughout the growing period, with supplementary irrigation applied during dry spells to ensure consistent moisture availability.

In order to determine the optimal basal fertilizer application rate and planting density affecting the yield of colored potatoes, three basal fertilizer application rate gradients and three planting density gradients were set up, and their cross-combination resulted in nine experimental combinations (**Table 1**). Compound fertilizer: N-P_2_O_5_-K_2_O = 15-5-25 (Boron ≥ 0.02%, Zn ≥ 0.02%, Mg ≥ 1.0%), total nutrient content ≥ 45%. The fertilizer was manufactured by Chongqing Fuyuan Chemical Co., Ltd. (Chongqing, China) and was uniformly applied as a basal fertilizer prior to planting. For each cultivation combination, mature tubers of uniform size were selected, cut into small pieces, and pooled to form composite samples. The resulting samples were then freeze-dried, ground into a homogeneous powder, and stored at -80°C until further analysis for physiological index determination and omics profiling.

**Table 1:** Table of different cultivation treatment conditions.

A (basal fertilizer application rate)	B (density)
0.12 Kg m^-2^	5.25 plants m^-2^
0.15 Kg m^-2^	6.00 plants m^-2^
0.18 Kg m^-2^	6.75 plants m^-2^

### 2.2 Determination of starch content

The starch contents were determined by anthrone colorimetry (Guo et al., 2024) using the biochemicals kit (NMKD0213, Norminkoda Biotechnology Co.,Ltd. Wuhan, China).

### 2.3 Determination of anthocyanins

The anthocyanins concentration is determined by PH-differential method (Ning et al., 2024) with an ultraviolet spectrophotometer at λ620 and λ650.The anthocyanins concentration was calculated using the Arnon equation:


$$\text{C}=((\text{A}530-\text{A}620)-0.1(\text{A}650-\text{A}20)/\text{ϵ}\times (\text{V}/\text{m})/\text{x}\cdot 106\times \text{M}\times 10^{-3})$$

Where, C is the anthocyanins concentration (ng/g), A the absorbance at the corresponding wavelength, V the volume of the extracting solution (mL), the m weight of the fresh leaves (g). the M of Anthocyanin molecular weight (287.24), the M of anthocyanin extinction coefficient (4.62×106).

### 2.4 Determination of crude protein

Determination of crude protein sampling using Kjeldahl nitrogen determination method (Yuan et al., 2024) reference to GB/T 6432-2018.

### 2.5 Determination of ascorbic acid

The ascorbic acid detection using red phenanthroline colorimetric method by the plant ascorbic acid content detection kit (Norminkoda Biotechnology Co., Ltd. Wuhan, China) (Sun et al., 2023), following the manufacturer’s instructions. In each experiment, about 0.1 g of tissues was taken, and 1 ml of extract was added for ice bath homogenization. The extraction technique was based on the reduction of Fe^3+^ to Fe^2+^ with ascorbic acid and the formation of a red chelate of Fe^2+^ with phenanthroline, which has a strong absorption capacity at 534 nm. The absorbance value is directly proportional to the ascorbic acid content in the reaction solution. 1 mg of ascorbic acid was added to 10 ml of extractive solution (5% trichloroacetic acid), and then dilute it to 10, 20, 40, 60, 80, 100 μg/mL to plot the calibration curve. ascorbic acid was quantified according to the absorption value of the sample at 534 nm. The results were expressed as mg of ascorbic acid equivalent per g fresh weight (mg/g FW). The analyses were performed in triplicate.

### 2.6 Sample selection for multi-omics analysis

To gain deeper insights into the molecular mechanisms underlying the optimal cultivation strategy, transcriptomic and metabolomic analyses were performed on tuber samples from treatments A1B1, A2B1, and A3B1. These treatments constitute a gradient of fertilization levels under a uniform planting density (B1), which demonstrated the highest yield potential in preliminary screening experiments (**Table 2**). By focusing on this comparative system, we enable a precise dissection of how fertilization regimes influence key metabolic pathways and gene expression regulatory networks under optimal growth conditions.

**Table 2:** Yield (kg/m^2^) of colored potatoes under various conditions.

Group	Yield (kg/m^2^)			Average (kg/m^2^)	CV
	I	II	III		
A1B1	33.80	34.10	33.40	33.77 ± 0.35	1.04%
A1B2	35.50	35.30	25.30	32.03 ± 5.83	18.20%
A1B3	27.50	34.20	27.90	29.87 ± 3.76	12.59%
A2B1	35.80	31.50	38.50	35.27 ± 3.53	10.00%
A2B2	29.00	28.30	39.10	32.13 ± 6.04	18.80%
A2B3	29.20	32.00	32.50	31.23 ± 1.78	5.70%
A3B1	36.40	30.60	32.30	33.10 ± 2.98	9.00%
A3B2	35.20	25.00	28.80	29.67 ± 5.15	17.35%
A3B3	38.90	28.60	31.70	33.07 ± 5.28	15.97%

“I, II, III” represent the treatment groups.

### 2.7 Isolation of total RNA and transcriptome analysis

#### 2.7.1 RNA extraction

RNA extraction: Total RNA was extracted from the maturity period tissue using TRIzol^®^ Reagent (Plant RNA Purification Reagent for plant tissue) according the manufacturer’s instructions (Invitrogen) and genomic DNA was removed using DNase I (TaKara). Then RNA quality was determined by 2100 Bioanalyzer (Agilent) and quantified using the ND-2000 (NanoDrop Technologies). Only high-quality RNA sample (OD260/280 = 1.8~2.2, OD260/230≥2.0, RIN≥6.5, 28S:18S≥1.0, >1μg) was used to construct sequencing library.

#### 2.7.2 Library preparation

Library preparation, and Illumina Hiseq xten/Nova seq 6000 Sequencing:Messenger RNA was isolated according to polyA selection method by oligo(dT) beads and then fragmented by fragmentation buffer firstly. Secondly double-stranded cDNA was synthesized using a SuperScript double-stranded cDNA synthesis kit (Invitrogen, CA) with random hexamer primers (Illumina). Then the synthesized cDNA was subjected to end-repair, phosphorylation and ‘A’ base addition according to Illumina’s library construction protocol. Libraries were size selected for cDNA target fragments of 300 bp on 2% Low Range Ultra Agarose followed by PCR amplified using Phusion DNA polymerase (NEB) for 15 PCR cycles. After quantified by TBS380, paired-end RNA-seq sequencing library was sequenced with the Illumina HiSeq xten/NovaSeq 6000 sequencer (2 × 150bp read length).

#### 2.7.3 Read mapping and quantification

The raw paired end reads were trimmed and quality controlled bySeqPrep (https://github.com/jstjohn/SeqPrep) and Sickle (https://github.com/najoshi/sickle) with default parameters. Then clean reads were separately aligned to reference genome with orientation mode using HISAT2 (http://ccb.jhu.edu/software/hisat2/index.shtml) software. Gene expression levels were quantified using StringTie (https://ccb.jhu.edu/software/stringtie/) based on the reference annotation, and transcript abundances were estimated as transcripts per million (TPM). [(Lee et al., 2021)].

#### 2.7.4 Differential expression analysis and functional enrichment

RSEM (http://deweylab.biostat.wisc.edu/rsem/) was used to quantify gene abundances. Essentially, differential expression analysis was performed using the DESeq2/DEGseq/EdgeR with Q value ≤ 0.05, DEGs with |log2FC|>1 and Q value <= 0.05(DESeq2 or EdgeR)/Q value <= 0.001(DEGseq) were considered to be significantly different expressed genes). In addition, functional-enrichment analysis including GO and KEGG were performed to identify which DEGs were significantly enriched in GO terms and metabolic pathways at Bonferroni-corrected P-value ≤0.05 compared with the whole-transcriptome background. GO functional enrichment and KEGG pathway analysis were carried out by Goatools (https://github.com/tanghaibao/Goatools) and KOBAS.

### 2.8 Metabolome analysis

Maturity period potato are freeze-dried and send to Norminkoda Biotechnology ((Norminkoda Biotechnology Co., Ltd. Wuhan, China)) for extraction and mass spectrometry testing.

PCA: Unsupervised PCA (principal component analysis) was performed by statistics function prcomp within R (www.r-project.org). The data was unit variance scaled before unsupervised PCA.

Differential metabolites selected: For two-group analysis, differential metabolites were determined by VIP (VIP > 1) and absolute Log2FC (|Log2FC| ≥ 1.0). VIP values were extracted from OPLS-DA result, which also contain score plots and permutation plots, was generated using R package MetaboAnalystR. The data was log transform (log) and mean centering before OPLS-DA. In order to avoid overfitting, a permutation test (200 permutations) was performed.

### 2.9 Statistical analysis

All the assays described above were repeated at least three times with three biological replicates. ANOVA was used to detect statistical differences, and the LSD of means was determined by Student’s test using SPSS Statistics software (IBM). Three independent repetitions were performed for all experiments. Statistical analyses were performed using one-tailed Student’s t test. Significant differences are noted as follows: * p< 0.05, * p < 0.01, * p< 0.001.

## 3 Results

### 3.1 Determination of optimal planting density and fertilizer application rate for potato

To explore the appropriate planting density and fertilizer application rate, a gradient-based experiment was conducted with different fertilizer application rates (A1, A2, A3) and different planting densities (B1, B2, B3). All combinations of the two treatment factors were tested and planted in three replicates arranged in different locations to minimize experimental error.

Results for determining the optimal planting density and fertilizer application rate for potato are shown in **Table 2**. Under a consistent basal fertilization system, yield decreased as planting density increased, with maximum yield achieved at a density of 5.25 plants m^-^². Under fixed planting density conditions, maximum yield was achieved at a basal fertilizer application rate of 0.15 kg m^-^².

Statistical analysis using one-way ANOVA revealed that there were no statistically significant differences in yield among the nine treatment combinations at the *p* < 0.05 level. This outcome is likely attributable to the inherent field variability commonly encountered in agricultural experiments, as evidenced by the high coefficients of variation (CV) observed in certain treatment groups (e.g., A1B2, A2B2, and A3B2). Nevertheless, treatment A2B1 (0.15 kg·m^-^² basal fertilizer; 5.25 plants·m^-^² density) achieved the highest mean yield (35.27 kg·m^-^²), representing an 18.9% increase compared to the lowest-yielding treatment (A3B2, 29.67 kg·m^-^²). When only fertilizer application rate was considered for maximizing yield, 0.15 kg m^-^² was optimal. When only planting density was considered for maximizing yield, 5.25 plants m^-^² was optimal.

### 3.2 The best qualities were observed under A2B1 conditions

**Figure 1:**
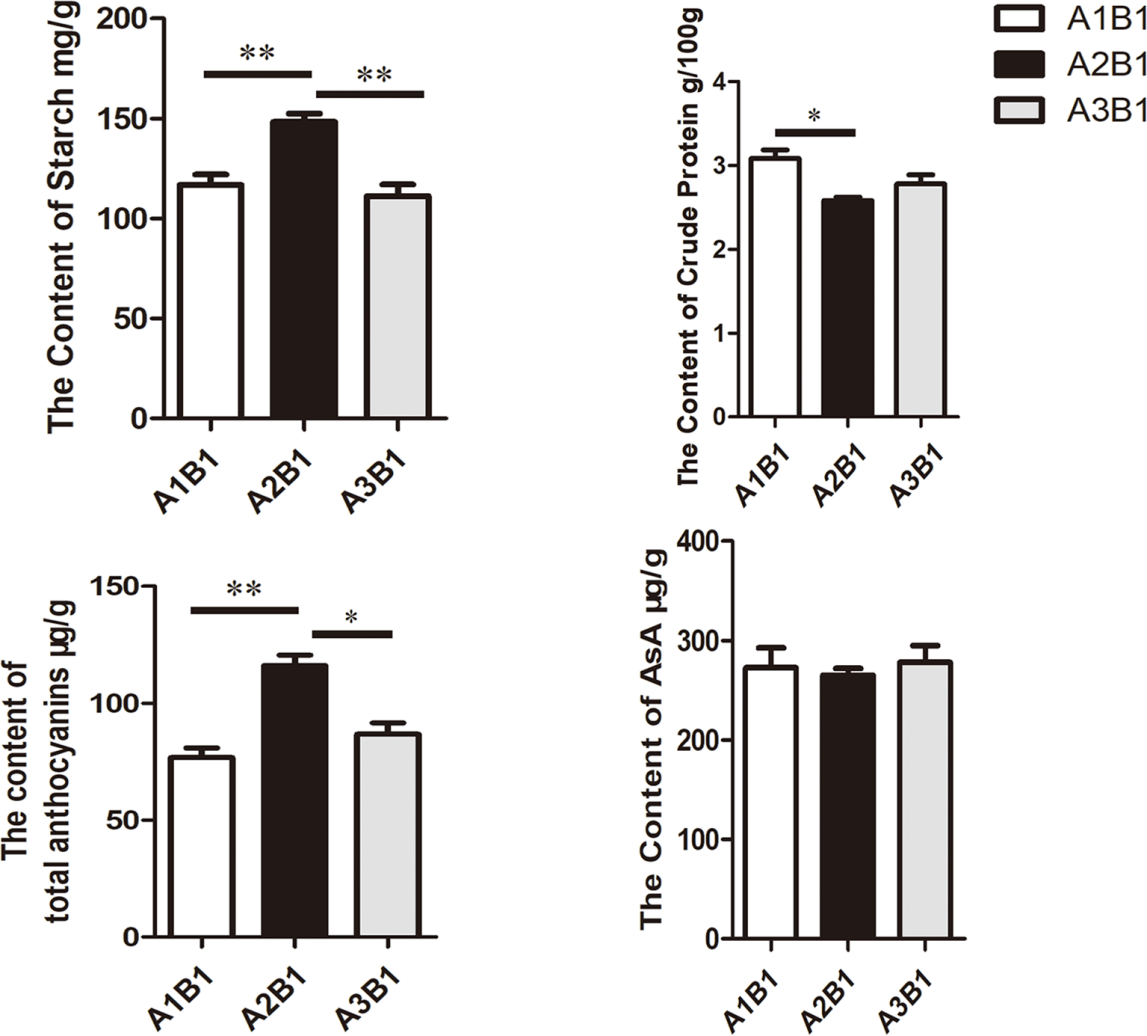
Quality evaluation of potato under diverse cultivation conditions. Data underwent a student’s test: * indicates *p* < 0.05, ** indicates *p* < 0.01. Bars=2 cm.

Quality parameters were assessed at consistent planting density with varying basal fertilizer application rates. The results are shown in **Figure 1**. Starch and total anthocyanin content reached maximum levels under A2B1, while crude protein content was minimal under this treatment (**Figure 1**). Moreover, compared with other experimental conditions involving different planting densities, purple potatoes exhibited the highest values for key quality parameters (anthocyanin and starch content) under the A2B1 treatment. **Figure 1** demonstrates synchrony between starch content, anthocyanin content, and yield, as seen in the yield line chart and nutrient content bar charts. However, there was no significant change in ascorbic acid. Pearson correlation coefficients between yield versus starch content and anthocyanin content across treatments were calculated. The results revealed that yield in the A2B1 treatment was maximized concurrently with starch (*r* = 0.92) and anthocyanin (*r* = 0.95) content.

Therefore, the combination of a basal fertilizer application rate of 0.15 kg m^-^² and a planting density of 5.25 plants m^-^² was the relatively best cultivation conditions for sweet potatoes in the mountainous areas of southern China.

### 3.3 Metabolome analysis of potato tubers under various conditions

To gain a clearer understanding of metabolite change patterns under different treatments, metabolites were identified using widely targeted metabolomics based on UPLC-MS. A total of 469 metabolites were detected, including flavonoids, alkaloids, amino acids and their derivatives, among others.

**Figure 2:**
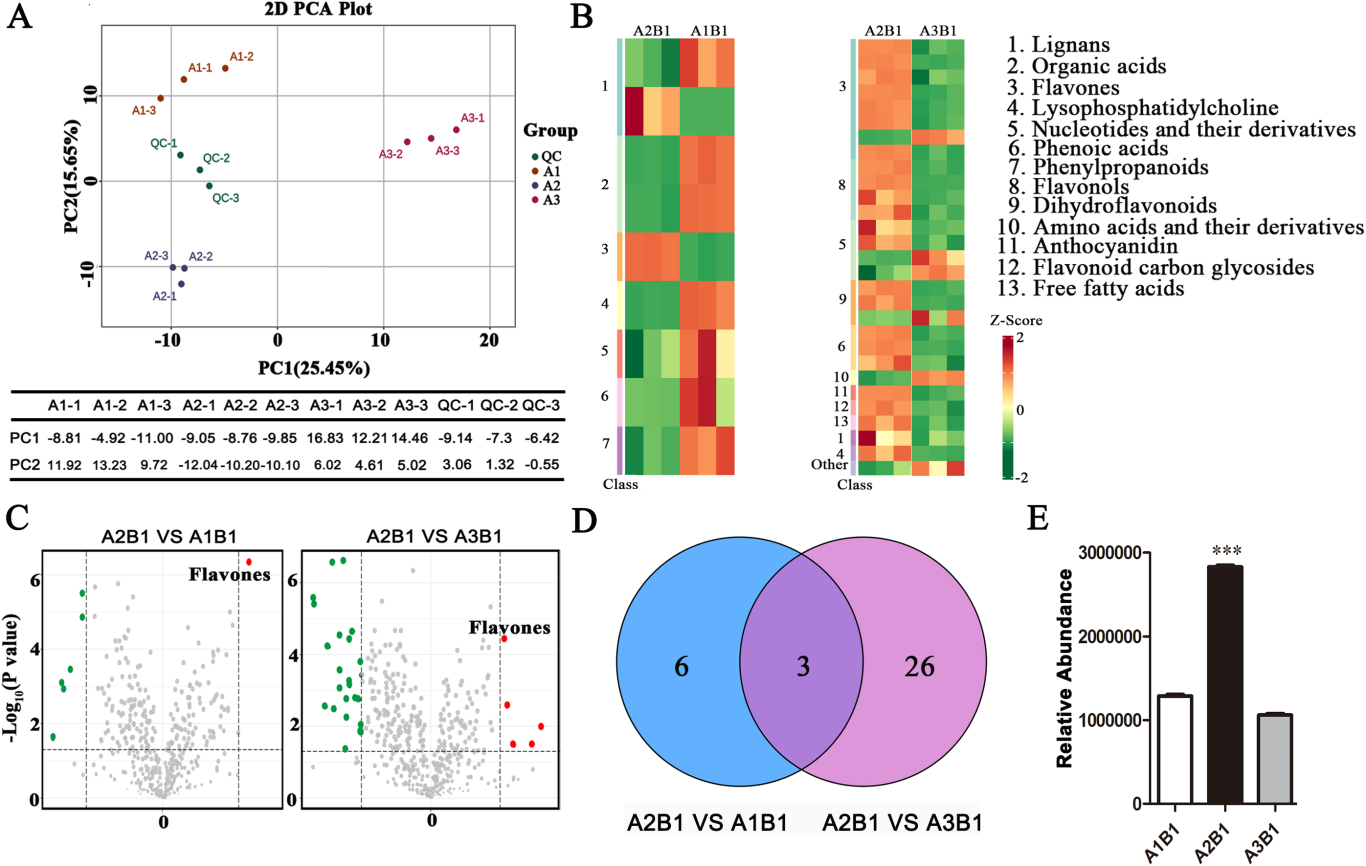
Analysis of metabolome quality and differential metabolite expression under different fertilizer application rates. **(A)** Principal component analysis (PCA) of DAMs; **(B)** Comparison of heat maps of DAMs between different groups; **(C)** The volcano plot shows the metabolites with increased and decreased abundance. Red dots, green dots and gray dots represent significantly increased, significantly decreased and non-significant metabolites respectively; **(E)** Analysis of Relative Abundance for flavones in groups; **(D)** Comparison of DAMs in groups on the Venn diagram. The overlap indicates the proportion of metabolites shared by each comparison group, while the non-overlap indicates the proportion of metabolites specific to the comparison group. Data underwent a student’s test: *** indicates p< 0.001, versus A1B1. Bars=2 cm.

The PCA analysis results show that the cumulative contribution rate of PC1 and PC2 is 41.1% (PC1: 25.45%, PC2: 15.65%), indicating that this model has a good data interpretation ability. As shown in **Figure 2A**, the distribution of the A2B1 group on the PC1 axis is significantly different from that of the other treatment groups, suggesting that its metabolic profile has significant uniqueness. Cluster heatmap analysis revealed differences in metabolite accumulation patterns across samples (**Figure 2B**). The KEGG classification map indicates that the differentially expressed genes are predominantly enriched in major functional categories, including “Metabolism,” “Genetic Information Processing,” and “Environmental Information Processing. Key metabolite classes included lignans, organic acids, flavonoids, lysophosphatidylcholines, nucleotides and derivatives, phenolic acids, phenylpropanoids, flavonols, dihydroflavonoids, amino acids and derivatives, anthocyanidins, flavonoid C-glycosides, free fatty acids, among others. Flavonoids were predominantly clustered within the A2B1 group.

Venn diagrams compared differentially accumulated metabolites (DAMs) across groups (**Figure 2C**). Three DAMs were shared between the comparison groups A1B1 vs A2B1 and A3B1 vs A2B1, comprising a flavonoid, a lignan, and a phenolic acid. Flavonoids were significantly accumulated in the A2B1 treatment (**Figures 2D, E**).

### 3.4 Transcriptome analysis of potato under various conditions

**Figure 3:**
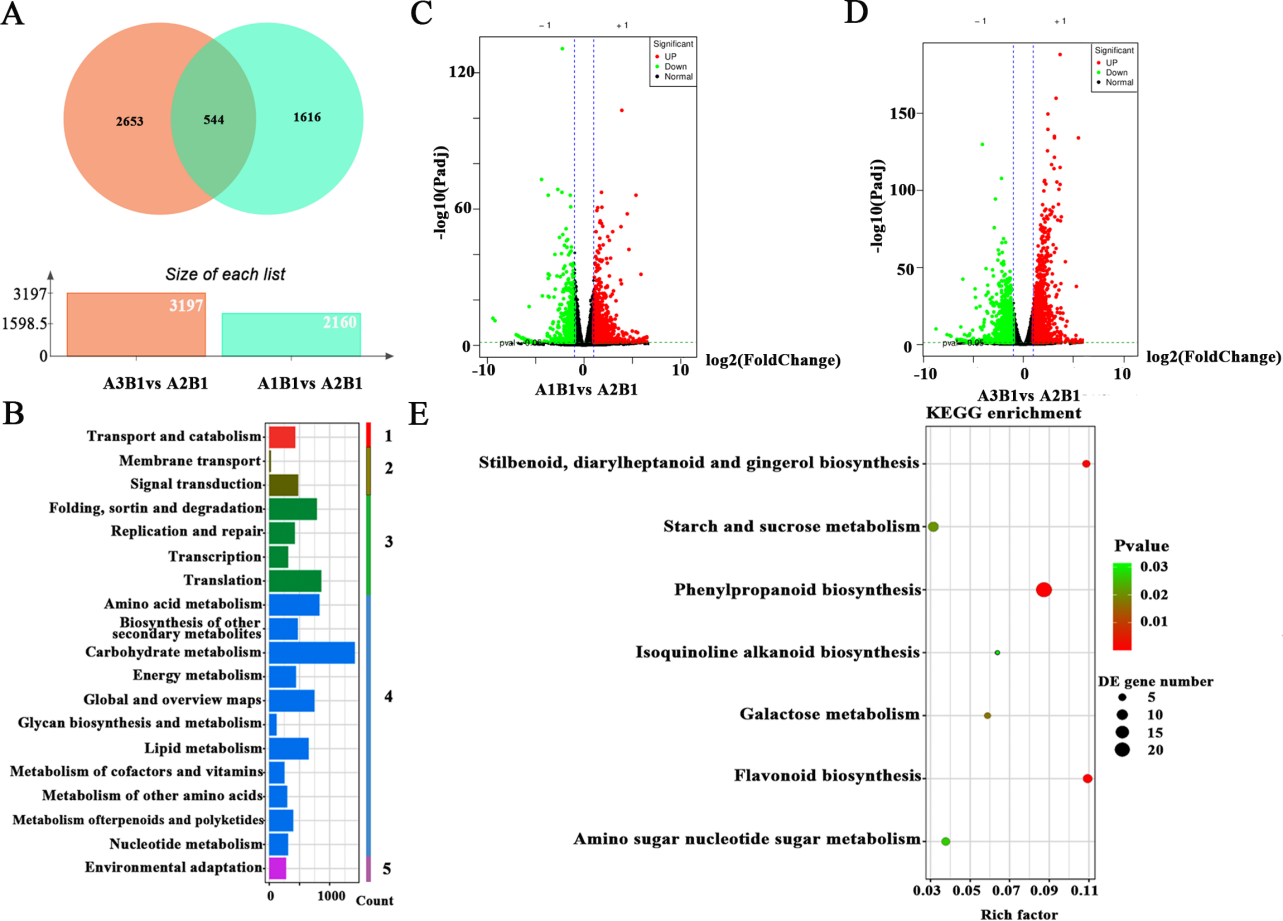
Transcriptome analysis of potato under different fertilizer application rates. **(A)** Venn diagram for the overlap between DEGs in each of the different treatments; **(B)** KEGG level 2 classification diagram of DEGs, 1 means cellular processes, 2 means environmental information, 3 means genetic information processing, 4 means metabolisms, 5 means organismal systems; C, **(D)** Volcano plots displaying the difference that are up-regulated and down-regulated genes between A2B1 and other treatment groups. Red points, green points and grey points indicate the genes that were significantly up-regulated, down-regulated and non-significance, respectively; **(E)** Differential expression gene KEGG pathway enrichment scatter plot.

Transcriptome analysis was performed on various treatment groups. The optimal treatment group (A2B1), exhibiting superior quality and yield, underwent differential gene expression analysis compared to other treatment groups. Analysis revealed 544 differentially expressed genes (DEGs) specific to the A2B1 group (**Figure 3A**). KEGG pathway enrichment analysis of these DEGs clarified their biological functions and associated pathways, including cellular processes, environmental information processing, genetic information processing, metabolism, and organismal systems (**Figure 3B**). Volcano plots visually represent distribution patterns of DEGs. For A2B1 vs. A1B1, analysis identified 2,160 DAMs (1,474 up-regulated, 686 down-regulated). Similarly, A2B1 vs. A3B1 showed 3,197 DAMs (1,499 up-regulated, 1,698 down-regulated) (**Figures 3C, D**). All DEGs met stringent thresholds (|fold change| ≥ 1 and VIP ≥ 1). The 544 DEGs common to both comparisons included key genes for flavonoid biosynthesis, starch and sucrose metabolism, among others (**Figure 3E**). These findings aligned with metabolomic results, indicating that genes involved in flavonoid biosynthesis and starch/sucrose metabolism significantly affected potato yield and quality across cultivation conditions.

### 3.5 Integrated transcriptome and metabolome analysis

**Figure 4:**
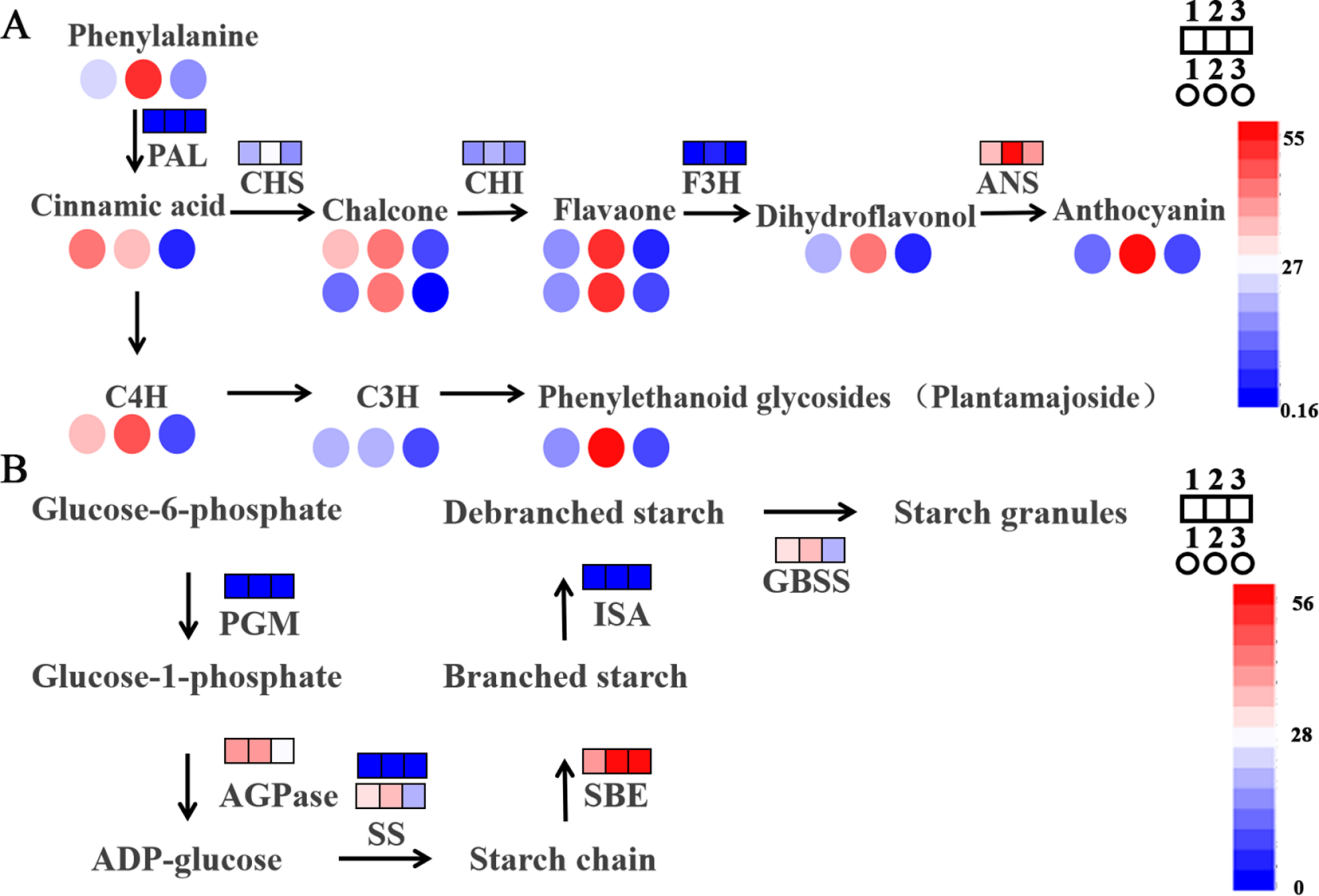
Major metabolic pathways of potato under different fertilizer application rates. **(A)** Contribution of DEGs and DAMs to flavones in potatoes under difference planting conditions; **(B)** Contribution of DEGs and DAMs to flavones in potatoes under difference planting conditions. 1 means A1B1, 2 means A2B1, 3 means A3B1. Square represents gene expression, circle represents metabolite abundance.

Combined with the transcriptome and metabolome analysis of this study, anthocyanin synthesis pathways (related to flavonoids) are mainly involved, so we conducted follow-up analysis (**Figure 4A**). Among these, many genes are differentially expressed in A2B1, such as dihydroflavonol reductase (DFR) (A2B1 is 5 times larger than A1B1, extremely significant; 2 times larger than A3B1, significant), anthocyanidin synthase (ANS) (A2B1 is 1.6 times larger than A1B1, highly significant; 1.4 times larger than A3B1, significant), UDP-glucose: Flavonoid 3-O-glucosyltransferase (UFGT) (A2B1 is 3.8 times larger than A1B1, highly significant; 4.5 times larger than A3B1, significant). These results show that there is a significant accumulation or expression of genes and metabolites related to the flavonoid and anthocyanin synthesis pathway under A2B1.

Additionally, genes differentially expressed in relation to starch and sucrose metabolism were also analyzed (**Figure 4B**), including starch synthase (SS) (A2B1 is 1.2 times larger than A1B1; 1.7 times larger than A3B1, extremely significant), starch branching enzyme (SBE) (A2B1 is 1.4 times larger than A1B1, highly significant; 0.9 times larger than A3B1), granule-bound starch synthase (GBSS) (A2B1 is 1.2 times larger than A1B1, highly significant; 1.7 times larger than A3B1, extremely significant), Isoamylase (ISA) (A2B1 is 1.1 times larger than A1B1; 2.1 times larger than A3B1, significant) and so on.

These results showed the importance of their impact on potato yield and quality under different cultivation conditions.

## 4 Discussion

### 4.1 Planting density and fertilization system are the key factors to improve potato quality

Potatoes (*Solanum tuberosum* L.) represent a globally significant food crop, and enhancing their yield and nutritional quality carries substantial practical implications. In conventional cultivation methods, planting density and fertilization practices emerge as primary determinants influencing crop productivity and quality (Liang et al., 2023). However, research on the relationship between purple potato yield and agronomic practices (including planting density and fertilizer application) remains scarce, limiting evidence-based optimization of cultivation protocols. This study focused on purple-fleshed potatoes to systematically evaluate the effects of gradient planting densities and basal fertilizer application rates on quality attributes (e.g., starch content, anthocyanin accumulation, crude protein levels). The objective was to establish agronomically optimized protocols for sustainable cultivation. Our study demonstrated that the A2B1 conditions (planting density, 5.25 plants m^-2^; fertilizer, 0.15 kg m^-^²) significantly enhanced tuber yield and quality. The yield increased by 12.9% compared with the minimum density treatment at the same fertilizer application rate, and increased by 6.6% compared with the minimum fertilizer application rate at the same fertilizer density. These results were consistent with previous researches indicating that balanced fertilization and density management are crucial for maximizing potato productivity (Getie et al., 2015; Assunção et al., 2021). In order to safeguard the fundamental yield for farmers, no fertilizer-free control group was established in this experiment. The fertilizer gradient design concentrated on the practical range (0.12–0.18 kg/m²) applicable in actual agricultural production. Nevertheless, the positive correlation between fertilizer application rates and crop yields observed in this study clearly substantiates the efficacy of fertilization. Under fixed fertilization regimes, yield was observed to decrease with increasing planting density, highlighting the necessity to avoid overcrowding as excessive density leads to resource competition and reduces tuber size (Tan et al., 2016). Conversely, optimal fertilizer application at 0.15 kg m^-^² likely ensured adequate nutrient availability for both vegetative growth and tuber bulking, a balance that is essential for achieving high yields (Yin et al., 2023).

### 4.2 Rational nutrient supply promotes starch accumulation and secondary metabolism in potato

**Figure 5:**
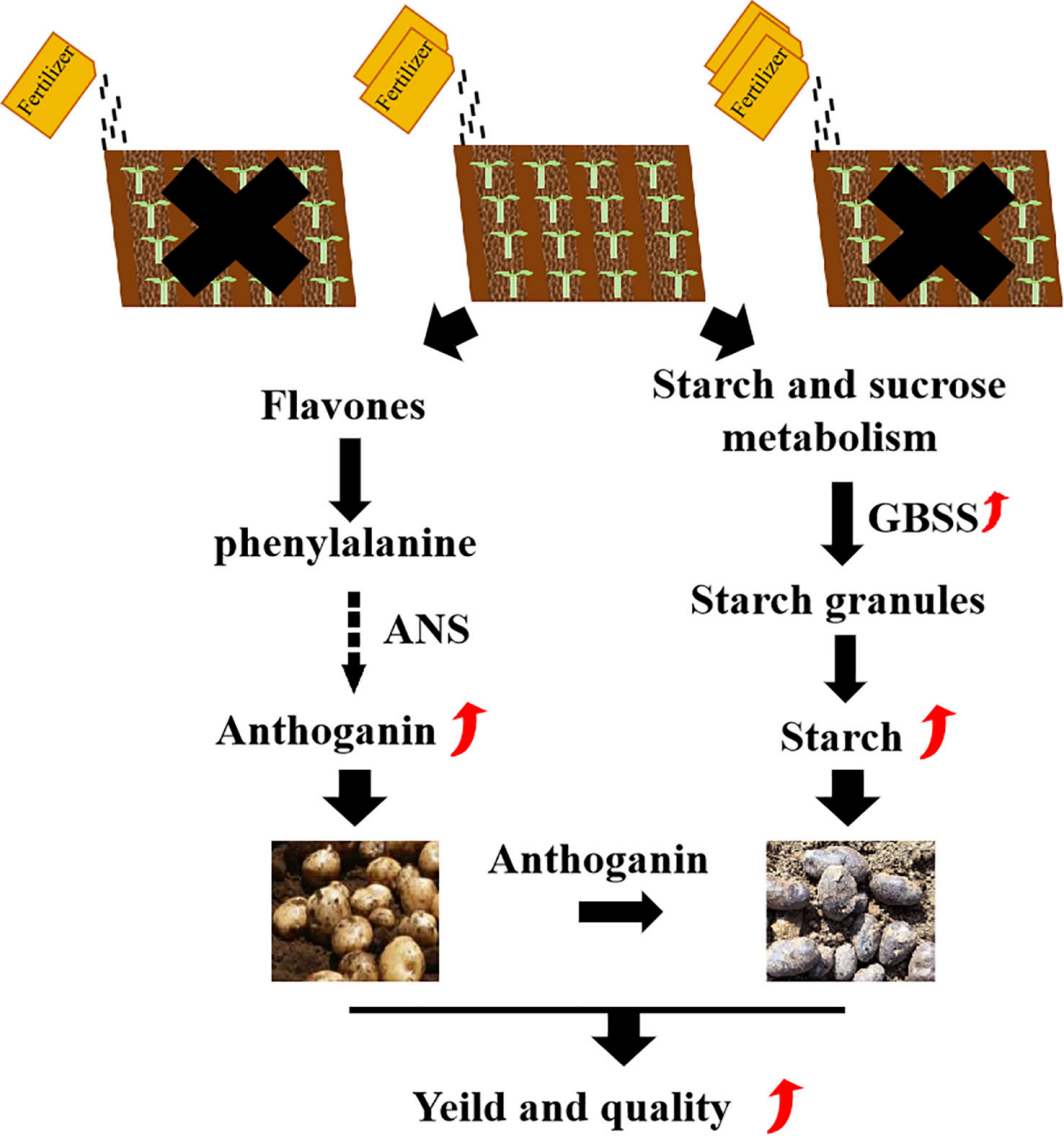
Modeling the impact of optimal planting density and fertilization levels on potato metabolism and transcriptional regulation.

The key quality traits of tubers at the mature stage (such as the final starch and anthocyanin content) tend to stabilize and reach their maximum values, which is a crucial time point for evaluating the final yield and quality. A large number of studies have shown that nutrients such as nitrogen, phosphorus and potassium have a continuous regulatory effect on the synthesis of starch and secondary metabolites throughout the growth period, especially during the tuber expansion period. The differences in gene expression can be detected at maturity. Compared to conventional white-fleshed potato, purple potato exhibit superior nutrient profiles (e.g., anthocyanins, antioxidants) and enhanced stress resistance (e.g., drought, disease) (Tang et al., 2023; Zhang et al., 2024). In this research, metabolome and transcriptome analyses of purple potatoes with different fertilizer dosage were conducted. The experimental model was shown in **Figure 5**, metabolomic analysis revealed that that A2B1 treatment enriched compounds related to enhanced stress tolerance and nutritional quality, such as flavonoids, lignans, and phenolic acids (Zahedi et al., 2020). Furthermore, transcriptomic profiling identified 544 differentially expressed genes (DEGs) in A2B1, which were mainly involved in starch and sucrose metabolism, flavonoid biosynthesis and environmental stress response, and were consistent with the metabolome data. Among them, the significant up-regulation of anthocyanin synthetase (ANS) and granule-bound starch synthetase (GBSS) genes in A2B1 was consistent with the increase of anthocyanin and starch content. High expression of starch metabolism genes (SBE) in A2B1 promotes starch accumulation, a property critical for potato processing and energy content (Zhang et al., 2017; Cheng et al., 2021). Previous studies have also confirmed that potassium fertilizer can promote starch synthesis by up-regulating the expression of AGPase and GBSS (Geigenberger, 2011; Gao et al., 2021). These studies indicate that the balanced NPK of A2B1 (18:7:25) enhances nitrogen assimilation (NR, upregulated nitrate reductase) and potassium-dependent crose transport (SUT1), promoting the synthesis of starch through AGPase and GBSS (Getie et al., 2015; Ning et al., 2024). Meanwhile, the availability of phosphorus activates MYB75, which is a transcription factor that promotes anthocyanin biosynthesis (ANS, DFR) by binding to the PAL promoter (Bhargava et al., 2010). This synergy avoids resource competition because excessive nitrogen in A3 treatment inhibits MYB75, transferring cyclic carbon to growth metabolites rather than defense metabolites.

In addition, enrichment of the flavonoid biosynthetic pathway suggests that A2B1 conditions may enhance the antioxidant capacity of potatoes, thereby improving tuber transport and mechanical damage (Devaux et al., 2021). We also observed the enrichment of gene pathways related to the synthesis of polyphenols and sugars. The co-expression of these pathways highlights the synergistic effects of optimized agronomic practices on yield and quality. These findings are confirmed by Sha et al (Sha et al., 2022), who report that balanced fertilization strategies increase starch and protein content while reducing sugars, thereby improving tuber quality.

However, the analysis results of crude protein and AsA content of purple potato showed that there was no positive correlation between purple potato yield and fertilizer application and planting density. Single-element fertilizers all can enhance potato yield, but exert differential effects on quality attributes: potassium fertilizers mainly increase starch yield (Guo et al., 2024), phosphorus fertilizers elevate flavonoids, anthocyanins, and carotenoid content (Santos-Silva et al., 2023), nitrogen fertilizers not only boost protein content but also modulate vitamin and flavonoid levels. Therefore, the content of crude protein and ASA in purple potatoes may be related to the type of fertilizer and the corresponding amount of application, which provides a direction for subsequent research.

### 4.3 Fertilization strategies are limited due to regional climate differences

This study experiment was conducted under controlled field conditions in southern China, and it remains uncertain whether these results are applicable to other regions with different soil types or climates. For example, Luo et al. emphasize that drought stress in northern China requires tailored irrigation strategies and fertilization (Luo et al., 2024). In addition, long-term field trials are needed to assess the sustainability of high fertilizer inputs, as excessive nitrogen application can lead to soil degradation and environmental pollution (Milroy et al., 2019; Sha et al., 2022; Jiang et al., 2025). Soil basic fertility is different in different regions, so controlling the proportion of NPK and other trace elements supplementation has a great influence on potato cultivation (Santos-Silva et al., 2023). Regarding the research materials, the focus of this study is Jinyun No. 5, as it has a high anthocyanin content and strong adaptability to the mountainous areas in southern China. However, this finding may not generalize to other purple sweet potato varieties with different genetic backgrounds. Future work will verify these practices in different varieties (such as Heimeiren, Ziyun) to ensure wider applicability. Our comprehensive approach combining agronomic, metabolomic, and transcriptomic analyses provides a robust framework for optimizing potato cultivation. It should be noted that this study was conducted within a single growing season under specific environmental conditions in the Chongqing region. Although our integrated omics approach provided strong mechanistic support for the observed phenotypic effects, the universality of the optimal cultivation parameters (0.15 kg of base fertilizer per square meter and a planting density of 5.25 plants per square meter) should be verified in future studies across multiple seasons and different geographical regions. Furthermore, the present study focused on the potato cultivar ‘Jinyun No.5’; whether the results can be extended to other purple potato varieties with distinct genetic backgrounds (e.g., ‘Heimeiren’, ‘Ziyun’) requires further validation.

## 5 Conclusions

This research focused on purple-fleshed potatoes to systematically evaluate the effects of gradient planting densities and basal fertilizer application rates on quality attributes (e.g., starch content, anthocyanin accumulation, crude protein levels) in the mountainous areas of southern China. Finally, it was determined that among all the experimental combinations, when the fertilization amount is 0.15 kg/m^2^ (N-P_2_O_5_-K_2_O=15-5-25) and the planting density is 56 plants/10.67 m², the purple potato has the best yield and quality. The program not only maximizes yields, but also improves nutritional and industrial quality, providing practical solutions for small-scale farming in resource-limited areas. In future research, we propose to validate the fertilization system and planting density examined in this study across other agricultural ecological zones (such as the arid regions of northern China), investigate the underlying molecular mechanisms to elucidate the trade-off between yield and quality, and incorporate multi-omics data into a precision agriculture prediction framework. In future research, we intend to validate the fertilization system and planting density investigated in this study across other agricultural ecological zones and over multiple growing seasons to confirm their stability and reliability. Delving deeper into the underlying molecular mechanisms will further elucidate the intricate trade-offs between yield and quality.

## Data Availability

The data presented in the study are deposited in the NCBI repository, accession number PRJNA1358814.
